# Minithoracotomy *vs*. Conventional Mitral Valve Surgery for Rheumatic Mitral Valve Stenosis: a Single-Center Analysis of 128 Patients

**DOI:** 10.21470/1678-9741-2019-0430

**Published:** 2020

**Authors:** Igor Chernov, Soslan Enginoev, Dmitry Koz’min, Gasan Magomedov, Dmitry Tarasov, Michel Pompeu B. O. Sá, Alexander Weymann, Konstantin Zhigalov

**Affiliations:** 1 Department of Cardiac Surgery, Federal Center for Cardiovascular Surgery, Astrakhan, Russia.; 2 Department of Cardiovascular Surgery, Astrakhan State Medical University, Astrakhan, Russia.; 3 Department of Cardiovascular Surgery, Pronto Socorro Cardiológico de Pernambuco - PROCAPE, Recife, PE, Brazil.; 4 Department of Thoracic and Cardiovascular Surgery, West German Heart and Vascular Center Essen, University Hospital of Essen, University Duisburg-Essen, Essen, Germany.

**Keywords:** Mitral Valve Stenosis, Mitral Valve-surgery, Mini-Thoracotomy, Coronary Artery Disease, Myocardial Ischemia, Prostheses and Implants, Hospitalization, Postoperative Complications

## Abstract

**Objective:**

To compare the in-hospital outcomes of a right-sided anterolateral minithoracotomy with those of median sternotomy in patients who received a mitral valve replacement (MVR) because of rheumatic mitral valve stenosis (RMS).

**Methods:**

This is a retrospective analysis of 128 patients (34% male) with RMS between 2011 and 2015. The median age was 53 years (45; 56). The mean ejection fraction was 58.4±6.3%. All the subjects were divided into two groups - Group 1 contained 78 patients who underwent MVR via minithoracotomy (MT-MVR), while Group 2 contained 50 patients who underwent MVR via median sternotomy (S-MVR).

**Results:**

In the MT-MVR group, a mechanical prosthesis was implanted in 72% of cases, while it was implanted in 90% of cases in the S-MVR group (*P*=0.01). The duration of myocardial ischemia was similar (MT-MVR, 77±24 min; S-MVR, 70±18 min) (*P*=0.09). However, the cardiopulmonary bypass time was lower in the S-MVR group than in the MT-MVR group (99±24 min and 119±34 min, respectively) (*P*≤0.001). There was no difference in the duration of mechanical ventilation, intensive care unit stay, and hospitalization period. Postoperative blood loss was lower in the MT-MVR group (*P*≤0.001) than in the S-MVR group. There are no statistically significant differences in postoperative complications (superficial wound infection, stroke, delirium, pericardial tamponade, pleural puncture, acute kidney insufficiency, and implantation of pacemaker). The overall in-hospital mortality was 3.9% (*P*=0.6)

**Conclusion:**

The minimally invasive approach for RMS is feasible and has an excellent cosmetic effect without increasing the risk of surgical complications.

**Table:** 

Abbreviations, acronyms & symbols	
AKI	= Acute kidney insufficiency
CI	= Confidence interval
CPB	= Cardiopulmonary bypass
ICU	= Intensive care unit
MT-MVR	= Mitral valve replacement via minithoracotomy
MV	= Mitral valve
MVR	= Mitral valve replacement
NYHA	= New York Heart Association
RMS	= Rheumatic mitral valve stenosis
S-MVR	= Mitral valve replacement via sternotomy
SD	= Standard deviation

## INTRODUCTION

Over the past few decades, minimally invasive surgery has revolutionized many aspects of the surgical treatment of mitral valve (MV) disease. Minimally invasive surgery is aimed at improving the cosmetic effect, reducing trauma, and a shorter period of hospitalization, while maintaining the safety and effectiveness of this access. Minimally invasive MV surgery using the videothoracoscopic approach was first introduced in the mid-1990s^[1,2]^. Since then, several studies have demonstrated the feasibility of minithoracotomy for MV interventions for selected patients in specialized high-volume centers^[3-6]^.

Rheumatic lesions of the MV remain the leading cause of mitral stenosis in endemic countries^[7]^. Only surgery (MV replacement or reconstruction) can be used to treat such patients. Nevertheless, the evidence for the use of mini access in such patients is insufficient.

In this article, we would like to share the experience of our clinic in the surgical treatment of rheumatic mitral valve stenosis (RMS). The aim of this study was to compare the immediate outcomes of a right-sided anterolateral minithoracotomy with those of sternotomy in RMS patients.

## METHODS

### Study Population

We present 128 patients with RMS who received mitral valve replacement (MVR) from 2011 to 2015 in our clinic. The median age of the patients was 53 years (45; 56). The studied population included 43 (34%) men; the preoperative mean left ventricular ejection fraction was 58.4±6.3%, with 95% confidence interval of 57-62%.

### Study Design

This study is a retrospective review of prospectively collected data. Data were collected as part of the institutional Mitral Valve Surgery Database and included detailed information on the patients’ demographics, baseline clinical characteristics, and their laboratory, echocardiographic, and hemodynamic parameters, as well as intraoperative variables and postoperative outcomes. The study was approved by the local ethics committee.

### Study Groups

All the subjects were divided into two groups. Group 1, 78 patients who underwent MVR via minithoracotomy (MT-MVR), and Group 2, 50 patients who underwent MVR via sternotomy (S-MVR). The choice of a surgical approach was based on the personal decision of a surgeon.

### Outcome Measures

The endpoints were operation time, cardiopulmonary bypass (CPB) time, aortic cross-clamp time, mechanical ventilation time, intensive care unit (ICU) stay, hospital stay, volume of drain blood loss, major complications (stroke, delirium, superficial wound infection, tamponade, pericardial effusion, pleural puncture, rupture of the left ventricular posterior wall, acute kidney insufficiency, implantation of pacemaker), and in-hospital mortality.

### Exclusion Criteria

- Redo procedure
- Hemodynamically significant coronary artery disease
- Concomitant cardiac surgery procedures
- Non-rheumatic MV disease

### Surgical Technique

Preoperatively, all the patients underwent ultrasound duplex scanning of the femoral vessels and computed tomography of the aorta. Introductory anesthesia and maintenance of anesthesia did not differ from standard heart surgery procedures. All the patients also underwent intraoperative transesophageal echocardiography before the skin incision and at the end of the operation. All the procedures were performed using CPB with normothermic perfusion and Custodiol cardioplegia. The peripheral CPB cannulation of femoral vessels was performed in MT-MVR patients and the central cannulation in the S-MVR group.

**Fig. 1:**
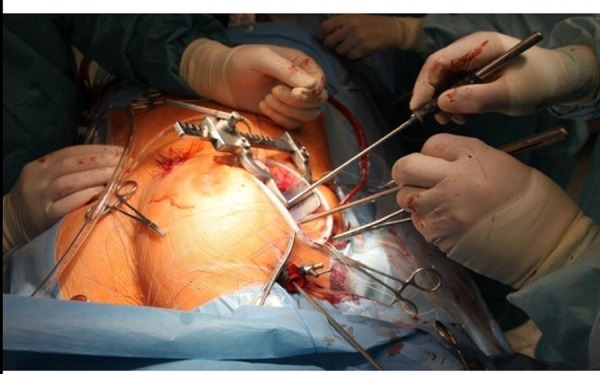
Mitral valve replacement via minithoracotomy: an access.

In the MT-MVR group, access to the heart was carried out from the right anterolateral minithoracotomy in the 4^th^ intercostal space (Figure 1). A video camera, an aortic clamp, and a hook for exposing the left atrium were inserted through separate punctures.

### Statistical Analysis

The data was analyzed using IBM SPSS Statistics software, version 25 (IBM Corp., Chicago, Illinois, United States of America). We used the Kolmogorov-Smirnov test to prove the data for normal distribution. Quantitative data was expressed as the mean and standard deviation for normally distributed variables and as the median and interquartile range for non-normally distributed variables. Categorical data was expressed as frequency and percentage. We used the Mann-Whitney U Test to compare the mean values and the Fisher’s exact test to examine the distribution of categorical variables between the groups. A value of *P*<0.05 was considered statistically significant.

## RESULTS

Demographic and preoperative clinical characteristics did not differ in both groups (Table 1). In all cases, MVR was performed because of the impossibility of the reconstruction of RMS. In the MT-MVR group, a mechanical prosthesis was implanted in 72% of cases; in the S-MVR group, it was implanted in 90% of cases (*P*=0.01). The type of prosthesis was selected regarding the guidelines and the patients’ preferences, depending on the possibility of taking warfarin and monitoring International Normalized Ratio levels.

**Table 1:** Demographics and preoperative clinical characteristics.

Variable	MT-MVR (Group 1, n=78)	S-MVR (Group 2, n=50)	*P*-value
Age (years), median (25 and 75 percentiles)	51 (44;56)	54 (50;56)	0.09
Gender (female:male)	50:28:00	35:15:00	0.56
Stroke, n (%)	4 (5,1%)	0 (0%)	0.15
NYHA III-IV class, n (%)	46 (59%)	35 (70%)	0.26
Pulmonary artery pressure (mmHg)	44 (38;50)	50 (37;60)	0,06
Left ventricular ejection fraction (%), median (25 and 75 percentiles)	59±5.3 (CI:58;60)	58±7.5 (CI:56;60)	0.5
Left atrial volume (ml), median (25 and 75 percentiles)	127 (96;162)	135 (105;170)	0,47
Atrial fibrillation, n (%)	37 (47.4%)	28 (56%)	0,37

CI=confidence interval; MT-MVR=mitral valve replacement via minithoracotomy; NYHA=New York Heart Association; S-MVR=mitral valve replacement via sternotomy

The total operation time and myocardial ischemia time did not differ in both study groups (*P*>0.05), while the CPB time was lower in the S-MVR group than in the MT-MVR group (*P*≤0.001). Intraoperative data is presented in Table 2. The duration of mechanical ventilation, ICU stay, and total hospital stay was similar in both groups. Postoperative blood loss was lower in the MT-MVR group than in the S-MVR group (*P*≤0.001). There were no statistically significant differences in postoperative complications (Table 3). We also did not observe any difference in mortality between the two study groups.

**Table 2:** Intraoperative variables.

Variable	MT-MVR (Group 1, n=78)	S-MVR (Group 2, n=50)	*P*-value
Mitral valve replacement, n (%)	78 (100%)	50 (100%)	-
Mechanical prosthesis, n (%)	56 (72%)	45 (90%)	0.01
Duration of the operation (min), mean±SD	179±41 (CI:170;189)	167±42 (CI:155;179)	0.1
Cardiopulmonary bypass time (min), mean±SD	119±34 (112;126)	99±24 (92;106)	≤0.001
Aortic cross-clamp time (min), mean±SD	77±24 (71;82)	70±18 (65;75)	0.09
Left atrium appendage closure, n (%)	8 (10.3%)	11 (22%)	0.08

CI=confidence interval; MT-MVR=mitral valve replacement via minithoracotomy; S-MVR=mitral valve replacement via sternotomy; SD=standard deviation

**Table 3:** Details of various postoperative complications.

Variable	MT-MVR (Group 1, n=78)	S-MVR (Group 2, n=50)	*P*-value
Myocardial infarction, n (%)	3 (3.8%)	0 (0%)	0.28
Stroke, n (%)	1 (1.3%)	3 (6%)	0.3
Pericardial effusion, n (%)	0 (0%)	3 (6%)	0.057
Mechanical ventilation time (hours), median (25 and 75 percentiles)	9 (7;12)	9 (7;12)	0.78
Volume of drain blood loss (ml), median (25 and 75 percentiles)	175 (125;231)	275 (213;350)	≤0.001
Tamponade, n (%)	1 (1.3)	0 (0%)	1
Delirium, n (%)	3 (3.8%)	2 (4%)	0.65
Reoperation, n (%)	3 (3.8%)	0 (0%)	0.28
Pacemaker, n (%)	2 (2.6%)	0 (0%)	0.52
Superficial wound infection, n (%)	1 (1.3%)	3 (6%)	0.3
AKI, n (%)	4 (5.1%)	5 (3.4%)	0.16
Rupture of left ventricular posterior wall, n (%)	1 (1.3%)	0 (0%)	1
Pleural punction, n (%)	9 (12%)	4 (8 %)	0.76
Intensive care unit stay (hours), median (25 and 75 percentiles)	20 (17;26)	22 (18;36)	0.38
Hospital stay (days), median (25 and 75 percentiles)	12 (10;14)	13 (11;15)	0.2
Mortality, n (%)	3 (3.8%)	2 (4%)	0.6

AKI=acute kidney insufficiency; MT-MVR=mitral valve replacement via minithoracotomy; S-MVR=mitral valve replacement via sternotomy

## DISCUSSION

**Fig. 2:**
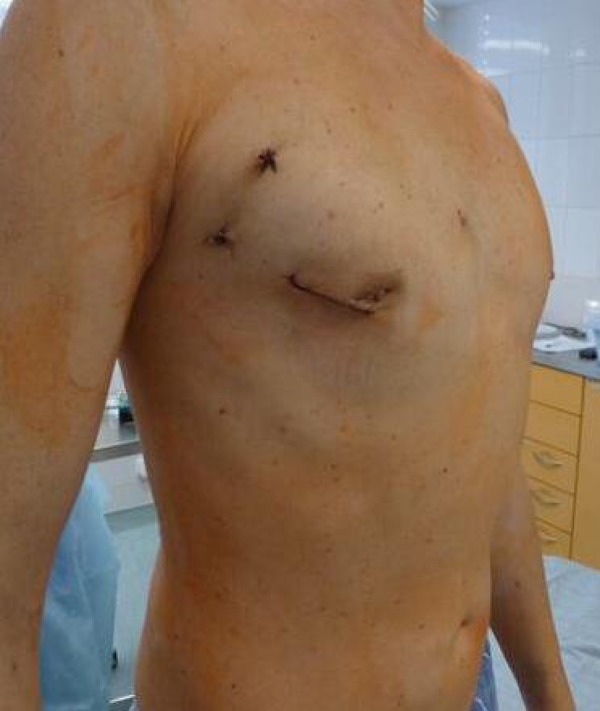
Mitral valve replacement via minithoracotomy: a final view.

In the mid-1990s, to minimize incision and trauma, various minimally invasive approaches were developed in MV surgery, including right parasternal approaches^[8]^ and superior and inferior hemisternotomy^[9]^. MT-MVR usually results in longer cross-clamp, CPB, and operative times. However, this fact does not affect the long-term survival and freedom of adverse events in MT-MVR compared with S-MVR^[10]^. Previous studies have reported the benefits of MT-MVR, including faster extubation; less postoperative pain, bleeding, and transfusion; better cosmetic results; and shorter duration of ICU and hospital stay (Figure 2) compared with S-MVR^[11-15]^. In contrast to these findings, we have not seen any difference regarding duration of ICU and inhospital stay.

**Fig. 3:**
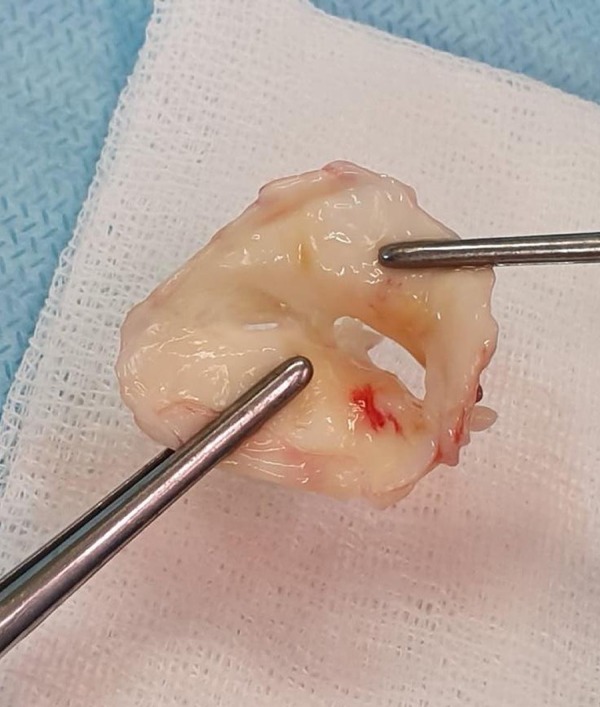
Excised rheumatic mitral valve.

There is still a lack of evidence regarding the use of the minimally invasive techniques for RMS. Chahal et al.^[16]^ published one randomized case-control study comparing right-sided minithoracotomy with sternotomy in patients with rheumatic MV lesions, during which it was shown that the minithoracotomy group had shorter ventilation time, hospitalization, and time spent in the ICU. The minithoracotomy group also experienced less bleeding, pericardial effusion, postcardiotomy syndrome, and blood transfusions and required less blood substitutes than the sternotomy group. In our study, we included patients with isolated MV disease. Only valve replacement was performed. Valve repair is also possible in patients with RMS and shows acceptable midterm results^[17]^. However, it depends on the severity of MV calcification (Figure 3). The long-term durability of the valve repair for RMS has been discussed^[7]^. We demonstrated a non-inferiority of MT-MVR compared with S-MVR in middleaged patients with RMS regarding survival and postoperative complications.

### Study Limitations

This study is a retrospective, nonrandomized analysis from a single medical center. The clinical decisions were made in a nonblinded fashion.

## CONCLUSION

The minimally invasive approach for RMS is feasible and has an excellent cosmetic effect without increasing the risk of surgical complications. A prospective randomized study on a large sample of patients is needed for more routine use of this technique.

**Table:** 

Authors' roles & responsibilities	
IC	Substantial contributions to the conception or design of the work; or the acquisition, analysis, or interpretation of data for the work; drafting the work or revising it critically for important intellectual content; agreement to be accountable for all aspects of the work in ensuring that questions related to the accuracy or integrity of any part of the work are appropriately investigated and resolved; final approval of the version to be published
SE	Substantial contributions to the conception or design of the work; or the acquisition, analysis, or interpretation of data for the work; drafting the work or revising it critically for important intellectual content; agreement to be accountable for all aspects of the work in ensuring that questions related to the accuracy or integrity of any part of the work are appropriately investigated and resolved; final approval of the version to be published
DK	Substantial contributions to the conception or design of the work; or the acquisition, analysis, or interpretation of data for the work; drafting the work or revising it critically for important intellectual content; agreement to be accountable for all aspects of the work in ensuring that questions related to the accuracy or integrity of any part of the work are appropriately investigated and resolved; final approval of the version to be published
GM	Substantial contributions to the conception or design of the work; or the acquisition, analysis, or interpretation of data for the work; drafting the work or revising it critically for important intellectual content; agreement to be accountable for all aspects of the work in ensuring that questions related to the accuracy or integrity of any part of the work are appropriately investigated and resolved; final approval of the version to be published
DT	Substantial contributions to the conception or design of the work; or the acquisition, analysis, or interpretation of data for the work; drafting the work or revising it critically for important intellectual content; agreement to be accountable for all aspects of the work in ensuring that questions related to the accuracy or integrity of any part of the work are appropriately investigated and resolved; final approval of the version to be published
MPBOS	Substantial contributions to the conception or design of the work; or the acquisition, analysis, or interpretation of data for the work; drafting the work or revising it critically for important intellectual content; agreement to be accountable for all aspects of the work in ensuring that questions related to the accuracy or integrity of any part of the work are appropriately investigated and resolved; final approval of the version to be published
AW	Substantial contributions to the conception or design of the work; or the acquisition, analysis, or interpretation of data for the work; drafting the work or revising it critically for important intellectual content; agreement to be accountable for all aspects of the work in ensuring that questions related to the accuracy or integrity of any part of the work are appropriately investigated and resolved; final approval of the version to be published
KZ	Substantial contributions to the conception or design of the work; or the acquisition, analysis, or interpretation of data for the work; drafting the work or revising it critically for important intellectual content; agreement to be accountable for all aspects of the work in ensuring that questions related to the accuracy or integrity of any part of the work are appropriately investigated and resolved; final approval of the version to be published
